# Editorial: Conditions and results of effective glycemic control in children with type 1 diabetes

**DOI:** 10.3389/fendo.2022.1034225

**Published:** 2022-09-22

**Authors:** Andrea Enzo Scaramuzza, Ivana Rabbone

**Affiliations:** ^1^ Division of Pediatrics, Pediatric Diabetes, Endocrinology and Nutrition, Azienda Socio Sanitaria Territoriale (ASST) Cremona, Cremona, Italy; ^2^ Division of Pediatrics, Department of Health Sciences, University of Piemonte Orientale, Novara, Italy

**Keywords:** glycemic control, type 1 diabetes, pediatric diabetes, long term complications, kidney disease, insulin pump therapy, continuous glucose monitor (CGM), technology

Glycemic control is important for reducing the risk of onset or progression of chronic complications in children with type 1 diabetes (1, 2). One of the most common complications in type 1 diabetes is diabetic nephropathy or diabetic kidney disease, which develops in 15-20% of individuals with type 1 diabetes (3). In this Research Topic, Mamilly et al. explore the role of ambulatory blood pressure monitoring and some urinary markers of tubular health and oxidative stress, including neutrophil gelatinase-associated lipocalin (NGAL), for the early detection of diabetic nephropathy. The authors also evaluate glycemic variability and determine that it might play a role in promoting kidney disease.

Children with type 1 diabetes and their parents or caregivers must become acclimatized to continuous self-management associated with avoiding complications. Glycemic control aims to effectively control blood glucose, blood pressure, and lipids in individuals with diabetes (Nwosu). Approaches include lifestyle changes to diet and exercise together with medications and insulin dosing. The challenges of glycemic control became even more acute during the COVID-19 pandemic, which has had a profound impact on patients with diabetes in terms of their health, morbidity, and mortality (4). Elbarbary et al. present their findings of a large global survey conducted during the pandemic that highlights that diabetes is more challenging to manage than any other pediatric endocrine disorder, with an increased risk of morbidity and need for intensive care in 21.2% of centers. A previous survey also found an increased risk of diabetic ketoacidosis at diabetes diagnosis (4, 5). In the survey, biopsychosocial concerns were common, including attempted suicide, highlighting the need to recognize and address psychological distress caused by COVID-19. The authors emphasize the importance of close contact with healthcare professionals and the ready availability of medical supplies to all patients, since over 20% of clinics faced a shortage of critical medications or supplies during the pandemic. However, many authors, including Nwosu et al., found no significant change or even an improvement (6–8) in glycemic control in patients with type 1 diabetes during the pandemic.

Over the last few years, new technologies have played an increasing role in glycemic control. By comparing multiple daily injections and insulin pump therapy, Wang et al. report that insulin pump therapy might be associated with better glycemic control and positively impact pediatric growth and development. Insulin pump users experienced no increase in risks of long-term complications and delayed pubertal development, providing evidence for an improved therapeutic regimen.

Although insulin pump therapy is an important treatment modality for patients with type 1 diabetes, pump use appears to vary internationally. Huo et al. investigated the application of insulin pump therapy in patients with type 1 diabetes in China and show that only a minority of patients use insulin pump therapy. When it was used, insulin pump therapy was associated with better blood glucose control and self-management. Patients with a younger age at diagnosis and longer duration of diabetes and patients with a higher socioeconomic status were more likely to use an insulin pump.

Guo et al. present a very interesting paper evaluating glycemic control of type 1 diabetes during the public Chinese New Year holiday in China. The Chinese New Year presents various challenges to glycemic control including increased stress, participation in social gatherings with high calorie foods and drinks, enjoyment of salty meals and alcoholic beverages, and limited opportunities to engage in physical activity. The data suggest that poor self-management may worsen glycemic control in the short term, indicating a need for more refined management algorithms during the holiday season for people with diabetes.

Psychological wellbeing is another cornerstone of diabetes management, as highlighted by the survey by Elbarbary et al. Two papers in this Research Topic evaluate the fear of hypoglycemia, usually the most important limiting factor to achieving metabolic control, and the psychological wellbeing of the parents of very young children with type 1 diabetes. In their paper, Glocker et al. explore psychosocial outcomes in young people with type 1 diabetes and their parents using currently available glucose monitoring devices in a real-life clinic setting. Parents were more likely to perceive higher levels of psychosocial burden related to their child’s diabetes than children and adolescents with type 1 diabetes, especially parents of younger children. This study highlights the need for family-based education and treatment resources to support parents in diabetes management in the face of rapidly advancing diabetes technology. In intermittently scanned CGM users, higher parental fear of hypoglycemia and lower parent-perceived quality of life correlated with a higher scanning frequency, indicating the potential impact of glucose monitoring on psychosocial outcomes or vice versa. The fear of hypoglycemia was especially associated with increased stress in parents of younger children, leading to frequent nocturnal blood glucose measurements and subsequent sleep deprivation, which in turn may affect parental well-being. Next steps in advancing technologies include the use of the artificial pancreas together with different algorithms. An initial evaluation of the use of the Cambridge algorithm in toddlers has demonstrated the feasibility of hybrid closed-loop insulin delivery in young children with diabetes (9), whereby parents reported reduced diabetes management burden and improved sleep quality (10). The paper by de Beaufort et al. presents data from baseline assessment with a specific focus on parental wellbeing in relation to fear of hypoglycemia. The authors report a higher score for hypoglycemia fear behavior compared with hypoglycemia worry. Parental wellbeing was negatively associated with hypoglycemia fear. They conclude that regular screening of parental well-being, hypoglycemia fear, and child behavior should form part of routine care to target early intervention.

Finally, for pediatricians, the transition of young adults (18-25 years old) from pediatric to adult diabetes centers can be challenging, and ~20% of patients experience a gap of over six months when moving from pediatric to adult care. Patients less prepared for transition are more likely to experience this gap. Care coordination and visit timing are important, as is technology to help transitioning. **Figure 1** summarizes of the updated pillars of type 1 diabetes management, together with an example of how technology can help and is rapidly becoming a new cornerstone of diabetes management.

**Figure 1:**
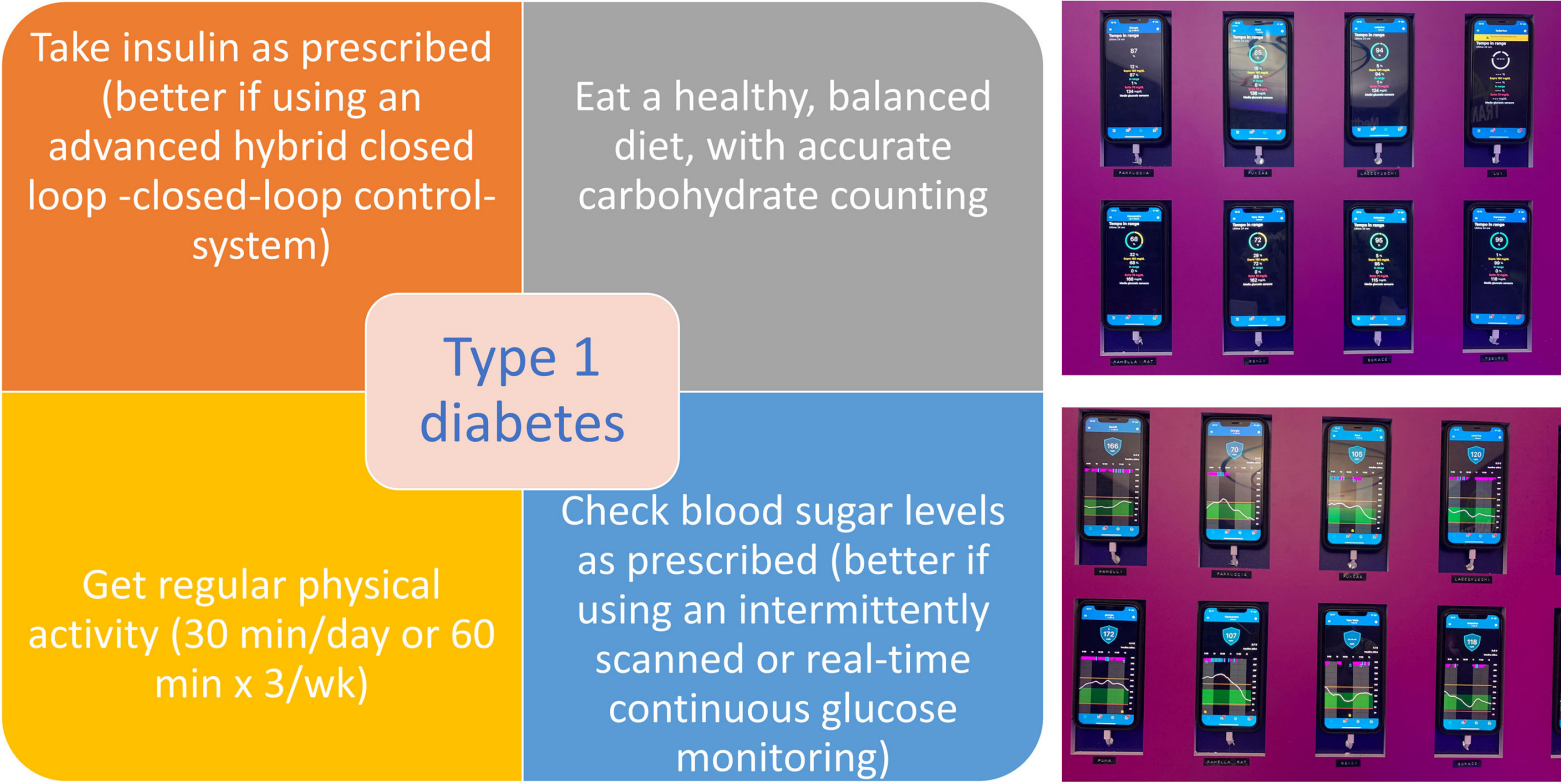
Updated type 1 diabetes management pillars (left), with an example of how technology can help. The continuous glucose monitoring profiles (right, bottom) and time in range percentages (right, top) of eight adolescents and young adults participating in the Transition Lab experiential training event held in Coccaglio, BS, Italy August 26-29, 2022.

## Author contributions

AS and IR conceived the Editorial, discussed it and wrote and approved the final version.

## Conflict of interest

The authors declare that the research was conducted in the absence of any commercial or financial relationships that could be construed as a potential conflict of interest.

## Publisher’s note

All claims expressed in this article are solely those of the authors and do not necessarily represent those of their affiliated organizations, or those of the publisher, the editors and the reviewers. Any product that may be evaluated in this article, or claim that may be made by its manufacturer, is not guaranteed or endorsed by the publisher.
